# QTL analysis of root morphology, flowering time, and yield reveals trade-offs in response to drought in *Brassica napus*

**DOI:** 10.1093/jxb/eru423

**Published:** 2014-11-04

**Authors:** Richard S. Fletcher, Jack L. Mullen, Annie Heiliger, John K. McKay

**Affiliations:** ^1^Department of Bioagricultural Sciences and Pest Management, Colorado State University, Fort Collins, CO 80523, USA; ^2^Cargill Specialty Seeds and Oils, Fort Collins, CO 80525, USA; ^3^Department of Soil and Crop Sciences, Colorado State University, Fort Collins, CO 80523, USA; ^4^Plant Genomics LLC, Fort Collins, CO 80524, USA

**Keywords:** *Brassica*, drought, pleiotropy, quantitative trait loci (QTLs), roots, trade-off.

## Abstract

Drought escape and dehydration avoidance represent alternative strategies for drought adaptation in annual crops. The mechanisms underlying these two strategies are reported to have a negative correlation, suggesting a trade-off. We conducted a quantitative trait locus (QTL) analysis of flowering time and root mass, traits representing each strategy, in *Brassica napus* to understand if a trade-off exists and what the genetic basis might be. Our field experiment used a genotyped population of doubled haploid lines and included both irrigated and rainfed treatments, allowing analysis of plasticity in each trait. We found strong genetic correlations among all traits, suggesting a trade-off among traits may exist. Summing across traits and treatments we found 20 QTLs, but many of these co-localized to two major QTLs, providing evidence that the trade-off is genetically constrained. To understand the mechanistic relationship between root mass, flowering time, and QTLs, we analysed the data by conditioning upon correlated traits. Our results suggest a causal model where such QTLs affect root mass directly as well as through their impacts on flowering time. Additionally, we used draft *Brassica* genomes to identify orthologues of well characterized *Arabidopsis thaliana* flowering time genes as candidate genes. This research provides valuable clues to breeding for drought adaptation as it is the first to analyse the inheritance of the root system in *B*. *napus* in relation to drought.

## Highlight text

This research is the first to examine the genetics of drought adaptive traits in *Brassica napus* and is supportive of an inherent trade-off between resource investment in reproduction and root development.

## Introduction

Nearly all aspects of terrestrial plant form and function depend upon adequate water availability. As a result, drought is the most common cause for reductions in crop yields, frequently causing reductions well below half of the crop’s theoretical yield potential (Boyer, 1982). A variety of mechanisms have been associated with drought acclimation (plasticity) and adaptation (heritable differences in traits) leading to the proposal of three distinct coping strategies (Ludlow, 1989): drought escape, dehydration avoidance, and dehydration tolerance. This report focuses on drought escape and dehydration avoidance, as dehydration tolerance is not prevalent in vascular plants, especially crops (Oliver *et al.*, 2010). A common strategy exploited in crop breeding is drought escape, which refers to plants that complete their life cycle prior to the onset of drought, thus avoiding moisture limitations. The alternative strategy, dehydration avoidance, is the sustaining of internal water status during dry external conditions by minimizing water loss and/or maximizing water uptake.

Resource limitation creates a necessity for organisms to allocate energy to processes in a competitive manner such that relationships among processes are constrained (Levins, 1968; Obeso, 2002). In plants there is a major energetic trade-off between investments in vegetative growth and reproduction, which can also be thought of as a life history trade-off (Reznick, 1985). Many studies have reported a trade-off between drought escape and mechanisms of dehydration avoidance, such as water-use efficiency and root size (Mitchell-Olds, 1996; McKay *et al.*, 2003; Wu *et al.*, 2010; Franks, 2011). However, results reporting the absence of such a trade-off (Sherrard and Maherali, 2006) suggest that more research is needed to understand the generality of this hypothesized constraint.

**Fig. 1.**
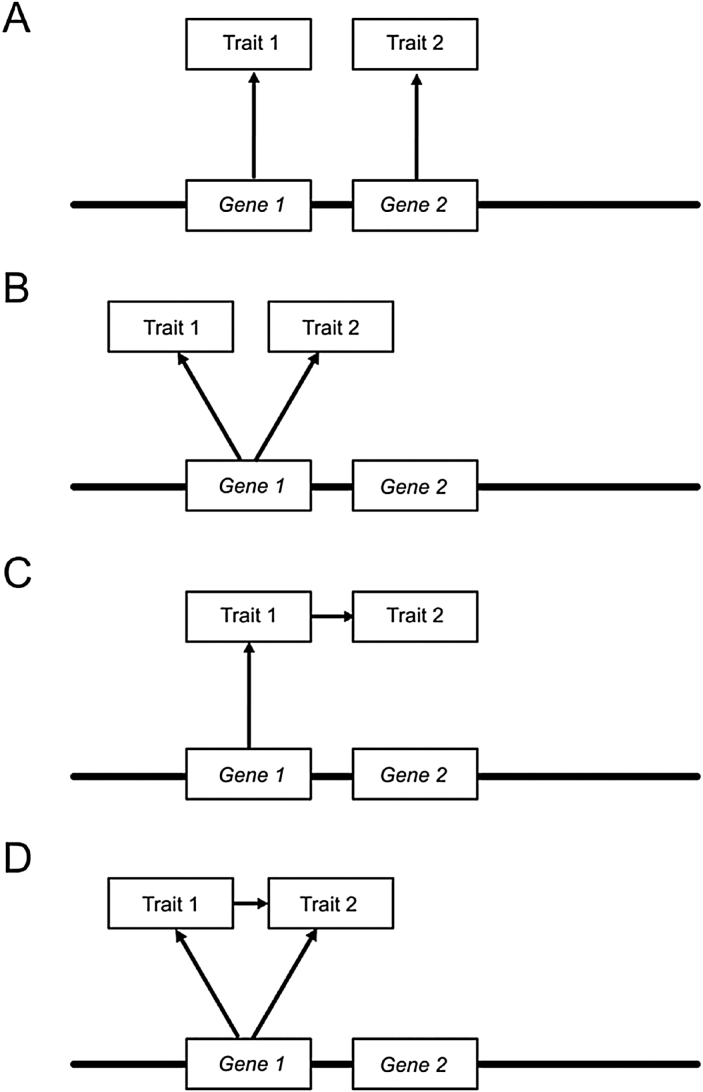
Diagram of putative mechanistic relationships; (A) genetic linkage; (B) pleiotropy; (C) physiological interaction; (D) combination of pleiotropy and physiological interaction.

Trade-offs can be quantified as genetic correlation coefficients, which measure the degree to which genetic variation in one trait predicts variation in the other (Robertson, 1959). Genetic correlations among traits can impose significant constraints on the efficacy and response to selection (both natural and artificial). This is because the adaptive optimum of trait values may be orthogonal to the vector of trait covariation. Genetically correlated traits are mechanistically the result of either genetic linkage or pleiotropy (Wagner and Zhang, 2011). In the case of genetic linkage (Fig. 1A), polymorphisms underlying variation at each trait are at different loci but are nearby physically, limiting recombination so that the trait value caused by the allele at one locus covaries with the trait value of the allele at the linked locus. Pleiotropy, on the other hand, refers to the effect an allele has on two or more phenotypes (Fig. 1B). Finally, genetic correlations may be due to physiological interactions among traits where one trait acts ‘upstream’ of another (Fig. 1C and D). Ultimately, genetic correlations due to pleiotropy constrain the response to selection far more than those due to genetic linkage (Futuyama, 1998).

In crops, drought escape is often achieved through breeding by optimizing flowering time. Flowering time marks the transition from vegetative to reproductive growth, and its influence on fitness and yield can be dramatic, making it perhaps the most important of all life history traits and the focus of extensive research in both crops and natural plant populations (reviewed in Michaels, 2009; Pose *et al*., 2012). The impact of flowering time on fitness may be due in large part to its many correlations with other diverse and potentially adaptive traits such as vegetative biomass (Shi *et al.*, 2009; Edwards *et al.*, 2012), vascular system development (Sibout *et al.*, 2008), oxidative stress (Kurepa *et al.*, 1998), water-use efficiency (McKay *et al.*, 2003; Franks, 2011) and a variety of root characteristics (Bolaños and Edmeades, 1993; Mitchell-Olds, 1996; Lou *et al.*, 2007). A study comparing isolines carrying mutant alleles in five loci annotated as ‘flowering time’ genes showed significant differences in morphological traits such as leaf length, leaf number, and auxillary shoot number, providing further evidence of pervasive pleiotropy at loci involved in flowering (van Tienderen *et al.*, 1996). The recurring association between flowering time and other traits is perhaps not surprising, since variation at any genes related to environmental sensing, resource acquisition, or resource allocation are also likely to lead to variation in flowering time (McKay *et al.*, 2008).

Dehydration avoidance is less well characterized, but mechanisms include reduced stomatal conductance and increased water uptake by roots. The root system has been long recognized as a central component of crop productivity (Sharp and Davies, 1979). This is due to the role of roots in water and nutrient acquisition, anchorage, mechanical support and, perhaps most importantly, sensing and responding to the complex and often heterogeneous soil environment. A dehydration avoidance strategy through maximization of water uptake clearly involves the root system, making it a focal subject of breeding for low rainfall environments (Ludlow and Muchow, 1990). Associations between drought adaptation and increased root system size and/or rooting depth have been drawn across many species (Cortes and Sinclair, 1986; Ekanayake, 1986; White and Castillo, 1989; Johnson *et al.*, 2000; Price *et al.*, 2001; Kirkegaard and Lilley, 2007; Lopes and Reynolds, 2010). However, the adaptive value of large or deep root systems varies by geography so that an applied breeding strategy must consider the climatic trends of the target production zone (Araus, 2002; Cativelli, 2008; Palta *et al.*, 2011). Selection for root traits is hindered by generally low heritabilities and the difficulty of phenotyping large populations (Wasson *et al.*, 2012; Topp *et al.*, 2013). Root traits remain a relatively unexploited breeding target, but additional insight into the genetic architecture of root system variation will be necessary for engineering ‘designer’ root systems to meet the world’s growing demand for food, fuel and fibre (Gregory *et al.*, 2013).

In this study, we investigated variation and covariation in drought escape and avoidance traits in *Brassica napus*. Of the *Brassica* oilseed crops, *B. napus* is the most important and trails only soybean and oil palm in terms of global production (http://apps.fas.usda.gov/psdonline/circulars/oilseeds.pdf; 2014). Changing climate conditions and expansion into new production geographies are increasing the exposure of *B. napus* crop production to drought stress. However, little research has focused on its root system, especially regarding inheritance, genetics, or relationship to drought.

We utilized a quantitative trait locus (QTL) mapping approach to better understand the genetic basis of root traits, flowering time, and their impacts on grain yield in *B. napus*. The QTL method is ideal for elucidating loci underlying trait correlations and, by including drought as an experimental treatment, loci associated with drought strategy trade-offs and yield sensitivity. We focused on a doubled haploid (DH) population of 225 lines derived from a cross between IMC106RR, an annual cultivar, and Wichita, a biennial cultivar, to maximize genetic and phenotypic diversity. The vernalization requirement differentiating annual (spring) and biennial (winter) lines also defines genetic and morphologically distinct pools (Diers and Osborn, 1994; Lühs *et al.*, 2003). We measured root pulling force (RPF, the vertical force required to remove a plant from the soil; Hayes and Johnson, 1939) as an index of root system size. Specifically, we identified a trade-off between flowering time and RPF, and revealed the genomic regions involved. In a more detailed follow-up study, we determined that variation in RPF is largely due to variation in taproot mass.

## Materials and methods

### Plant materials

This study utilized a DH population of 225 lines named SE-1 that was produced from an F1 generation microspore donor plant derived from a cross made between the annual variety IMC106RR (Cargill Inc., National Registration No. 5118), and the biennial variety Wichita (Rife *et al.*, 2001; Reg. no. CV-19, PI 612846) at Cargill (Fort Collins, CO, USA) using the method of Palmer *et al.* (1996). The resulting population segregated for the requirement of vernalization to initiate flowering and consisted of approximately 1200 lines. From this, about 900 lines flowered in the greenhouse and, thus, were deemed to have an annual growth habit. Of these 900 DH lines, we randomly selected 225 for use in this experiment.

### Genotyping and mapping

Genotyping was done using the Illumina (San Diego, CA, USA) *Brassica* 60K Infinium array at DNA Landmarks (Quebec, Canada). The final list of 1179 markers used in linkage map construction was selected based on GenTrain genotype scores above 0.75 as suggested by Illumina followed by selection for those which lack an inter-homeologous polymorphism (Trick *et al.*, 2009). The genetic linkage map was constructed in JoinMap3 (Van Ooijen *et al.*, 2001) using a threshold recombination frequency of <0.25 and a minimum logarithm of the odds ratio (LOD) score of 6 for grouping loci into linkage groups. The Kosambi function (Kosambi, 1944) was used to calculate genetic distances. Each linkage group was named based on the nomenclature recommended by the Multinational *Brassica* Genome Project steering committee (http://www.brassica.info/resource/maps/lg-assignments.php). The map was analysed further in the R/qtl program of the R statistical package (Broman *et al.*, 2003; Broman and Sen, 2009) to confirm marker orders and assess general map quality.

### Field design

The DH lines and parents were planted at Colorado State University’s Agricultural Research Education and Demonstration Center (40.66°N/105°W) near Fort Collins, CO, USA on 19 April 2010. The study was arranged in a Row-Column design (created with CycDesigN 3.0, www.cycdesign.co.nz) with three replicates per treatment. Plots comprised two rows separated by 0.23 m and were 1 m in length. Plots were separated by a distance of 0.33 m and thinned to ~10 plants per plot. Irrigation was applied using a linear-move system at approximately 2.5cm per week for the first month of development at which point it was discontinued in the rainfed (dry) treatment. Irrigation was maintained at the rate of 2.5cm per week for the duration of the experiment in the irrigated (wet) treatment.

### Phenotyping

Days to flower (DTF) was recorded for each plot as the interval from sowing date to the date on which 50% of the plants in the plot had initiated flowering. To measure yield, plots were swathed by hand, allowed to dry, threshed using a Wintersteiger (Wintersteiger AG, Austria) combine harvester and weighed immediately thereafter. Yield sensitivity was calculated as the difference between the yield of a DH line in the wet environment and its yield in the dry environment:

Yield (wet)–Yield(dry)

Relative yield was calculated as a *Z* score to account for the large differences in mean yield and standard deviation between the wet and dry treatments. The RPF method designed and used extensively in maize (Hayes and Johnson, 1939; Spencer, 1940; Rogers *et al.*, 1976; Lebreton *et al.*, 1995) was modified in a manner suitable for *B. napus*. In short, a lasso was formed from a nylon rope and harnessed around the base of a single *B. napus* plant within a field plot. A loop was formed at the other end of the rope and used to attach it to a hand-held Imada DS2 dynamometer (Imada Inc., Northbrook, IL, USA). The dynamometer was then slowly pulled vertically until the root system came completely out of the soil, and the maximum force (Kgf) generated during the root system removal was recorded. RPF was measured within 1–2 days after grain was harvested. To optimize the phenotype, the field was watered 24 hours before measuring RPF.

### Quantitative genetic analyses

A linear mixed model was used to analyse the data using the PROC MIXED procedure in the SAS software package (SAS Institute) with the DH line treated as a fixed effect and row and/or column treated as random effects. The broad-sense heritability (H^2^) for each trait was estimated using variance components computed in the PROC VARCOMP procedure in SAS as the ratio:

$$V_{G}:(V_{G}+V_{E})$$

where *V*_*G*_ is the variance among DH lines and *V*_*E*_ is the residual variance. Among-trait phenotypic correlations were computed as Pearson correlation coefficients using data points collected from individual plots and genetic correlations were computed from least square means estimated for each DH line within each environment (Falconer and Mackay, 1996).

QTL mapping was performed using Haley-Knott Regression (Haley and Knott, 1992) in R/qtl using 1 cM steps. QTLs were selected using a step-wise model selection approach (Manichaikul *et al.*, 2009) based on significance thresholds made from 1000 permutations (Churchill and Doerge, 1994). Genome-wide scans for QTL by environment interactions were conducted by comparing a model including the environment (moisture treatment) as a covariate along with a QTL–environment interaction to a model lacking the interaction. LOD 1.5 confidence intervals were determined in the R/qtl software package (Broman *et al.*, 2003; Broman and Sen, 2009). QTLs were named using the trait and treatment with which they were associated with ascending numbers based on linkage group location.

### QTL confirmation in the field

In an effort to validate the effect of a QTL discovered in this study (*RPF.dry1*) and to gain a better understanding of the mechanisms underlying RPF, a second study was performed during the summer of 2012. It focused on a total of 40 lines of which each parental haplotype at *RPF.dry1* was represented by 20 lines. Haplotypes were defined by DH lines which carried marker alleles spanning the LOD 1.5 confidence interval. The physical position of this interval was determined by comparing SNP sequence information with the *B. rapa* reference (Wang *et al.*, 2011; Cheng *et al.*, 2011) using BLAST (Altschul *et al.*, 1990). The experiment was conducted at the same experimental farm in which each experimental unit consisted of a single plant per DH line watered by a precision drip irrigation emitter. Three randomized blocks were planted and thinned to a single plant 2 weeks after germination. All other factors of the experiment were conducted as they were in 2010. In addition to collecting data on RPF and DTF, aboveground biomass was weighed for each plant in the field as they were extracted and shoot fresh weight (SFW) was also recorded. Root mass extracted during RPF measurement was oven dried at 80°C for 3 days prior to measurement of the taproot dry mass, lateral root dry mass, total root dry mass, tap root diameter (diameter of the basal portion of the dried taproot), tap root length (length of the extracted taproot), branching zone length (length of the taproot with primary laterals), and the number of coarse secondary roots (total number of secondary roots >1mm in diameter).

## Results

### The 19 chromosomes of *B. napus* are recovered in the genetic map

The genetic map recovered 19 linkage groups which represent the 10 chromosomes of the A genome (*B. rapa*; 2*n* = 20) and the nine chromosomes of the C genome (*B. oleracea*; 2*n* = 18) which comprise the allopolyploid genome of *B. napus*. The map was constructed using 1179 markers and resulted in a total length of 2041 cM, had an average intermarker distance of 1.8 cM, and carried large gaps of 46 cM and 33 cM on A01 and A08, respectively (Supplementary Figure S1). On average, segregation in the population met the expected 1:1 ratio (48.4% Wichita allele, 51.6% IMC106RR allele). Several regions showed segregation distortion in favour of the alleles from the Wichita parent, including most of linkage groups A01 and A08 with maximum biases of 66.2% (chi-square = 24.4) and 66.1% (chi-square = 24.1), respectively. This segregation in favour of alleles from the winter parent occurred despite selection for lines lacking a vernalization requirement and is probably the product of gametic selection during the microspore culture process (Ferrie and Möllers, 2011). Other regions of lower segregation distortion were also observed on A06 and C03 in favour of the IMC106RR allele and on C01 in favour of the Wichita allele. This minor amount of segregation distortion did not have a noticeable impact on map construction or QTL analysis (Hackett and Broadfoot, 2003).

### RPF, DTF, and yield demonstrate strong genetic correlations

A significant treatment effect (Supplementary Table S1) and heritable variation for RPF, DTF, and yield was observed. Much of this variation was attributable to genetics with estimates of heritability ranging from 0.16 for RPF in the dry treatment to 0.83 for DTF in the wet (Table 1). In agreement with previous research (Udall *et al.*, 2006; Shi *et al.*, 2009), flowering time had the highest heritability estimates in both treatments. Despite selecting initially against the vernalization requirement, some lines didn’t flower in the dry treatment resulting in a right-censored distribution (Leung *et al.*, 1997). Those DH lines that flowered did so in a minimum of 59 days in both treatments and a maximum of 101 days in the dry treatment and 108 days in the wet treatment. RPF and yield demonstrated transgressive inheritance in the DH population in both treatments, a parameter that could only be measured relative to IMC106RR for DTF and yield since Wichita, a winter growth-habit line, neither flowered nor set seed during the field season.

**Table 1.** Descriptive statistics for DTF, RPF, and yield measured in the SE-1 population in Fort Collins, CO, USA in 2011

Trait	Treatment	*N*	Mean	*SD*	Min	Max	H^2^
DTF	Wet	643	77.00	9.49	59	108	0.83
	Dry	544	75.05	7.79	59	101	0.73
RPF	Wet	651	36.71	20.65	1.9	127	0.25
	Dry	650	30.78	16.49	4.5	114.4	0.16
Yield	Wet	663	40.73	46.17	1	305	0.21
	Dry	667	6.90	9.35	0	55	0.53

**Fig. 2.**
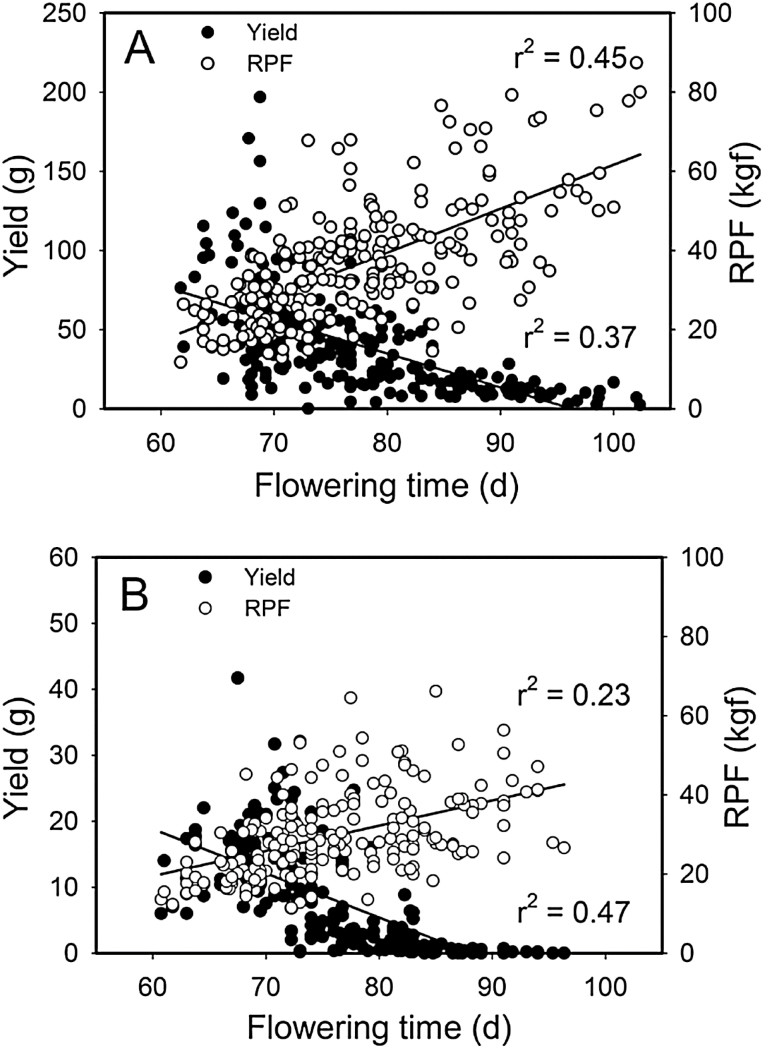
Genetic correlations among traits in the wet (A) and dry (B) treatments in the SE-1 population (*n* = 195–225; *P* < 0.0001).

Correlations were highly significant (*P* < 0.0001) among all traits (Fig. 2). Yield had a strong negative genetic correlation with both DTF and RPF under both treatments. Positive genetic and phenotypic correlations were observed between RPF and DTF so that late-flowering lines required a larger force for removal (Supplementary Table S2).

### QTL analysis identifies two major pleiotropic factors underlying the trade-off between drought escape and avoidance

**Fig. 3.**
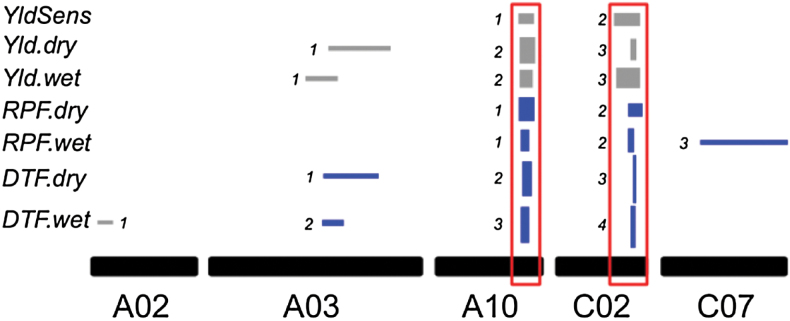
Localization and relative effect sizes of QTLs for the six traits analysed. Box widths indicate LOD 1.5 confidence intervals for the QTLs. The box height represents the percentage variance explained. Colour indicates the directional effect of the Wichita allele (blue, positive; grey, negative). The pleiotropic QTLs on chromosomes A10 and C02 are bracketed in red. Numbers next to boxes indicate the QTL naming scheme.

We scanned for QTLs associated with DTF, RPF, and yield along with the sensitivity of yield to drought. Seven QTLs for DTF were mapped; four in the wet and three in the dry treatments. For RPF, three QTLs were identified in the wet environment and two in the dry. Analyses of yield found three QTLs for each environment. Summing across all four traits, a total of 20 additive QTLs were discovered (Fig. 3). QTLs co-localized to regions on linkage groups A03, A10, and C02 (Fig. 3), thus implicating tight linkage or pleiotropy as the cause of the strong genetic correlations observed among traits. In particular, two regions on A10 and C02 (bracketed in red in Fig. 3) explained a large proportion of the variation for each trait and their estimated effects were always larger than other QTLs discovered for any particular trait. All of the QTLs discovered for yield co-localized with QTLs for DTF, further supporting the strong relationship between these traits where a flowering time of ~68 days increases the probability of higher yield (Fig. 2). These results also show that, unlike yield, the genetics underlying DTF and RPF did not overlap entirely. For example, *DTF.wet1* and *DTF.wet2* had no relationship to RPF where *RPF.wet3*, located on C07, had no relationship to DTF. This also suggests that the QTLs on A10 and C02 may be responsible for most, or all, of the genetic correlation (*r* = 0.45) of RPF measured in the wet and dry treatment (Supplementary Table S2).

**Fig. 4.**
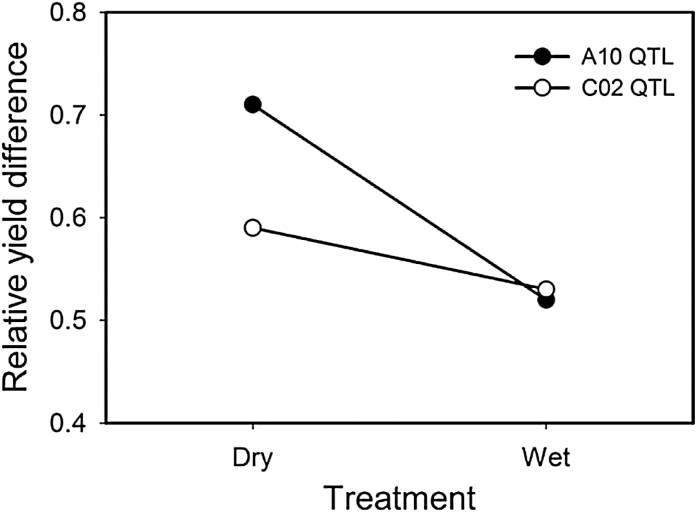
Difference between alleles (IMC106RR-Wichita) for relative yield (Z score) at QTLs on A10 and C02 under wet and dry treatments.

Analysis of variance showed the genotype by environment interaction to be significant for flowering time and yield (Supplementary Table S1), but significant QTL by environment interactions were only found for yield on chromosomes A10 and C02. The impact of the QTLs on A10 and C02 in response to treatment was further supported by QTLs which mapped to these two chromosomal locations for yield sensitivity (Fig. 3; a detailed summary of all QTL results is provided in Supplementary Table S3). A closer examination of the allele effects at each locus shows that late-flowering QTL alleles have a larger impact in the dry treatment than the wet (Fig. 4).

### Examination of the relationship between RPF and DTF using conditional QTL models shows evidence that *RPF.dry1* may be acting directly on both traits

To better understand the genetic architecture between the traits, the data were analysed with QTL models which conditioned upon flowering time, the hypothetically ‘upstream’ trait. The goal of this additional analysis was to infer the causal relationships among traits which share QTLs (Li *et al.*, 2006; Broman and Sen, 2009). More specifically, the objective was to elucidate whether a particular QTL is affecting a trait directly (Fig. 1B), as a downstream effect of delayed flowering (Fig. 1C), or a combination of both (Fig. 1D).

All of the QTLs for yield under both treatments disappeared when DTF was included as a covariate in the QTL scan (data not shown). This supports the intuitive notion that DTF is an upstream determinant of yield and the co-localizing QTLs act indirectly on yield via their effects on flowering time.

**Fig. 5.**
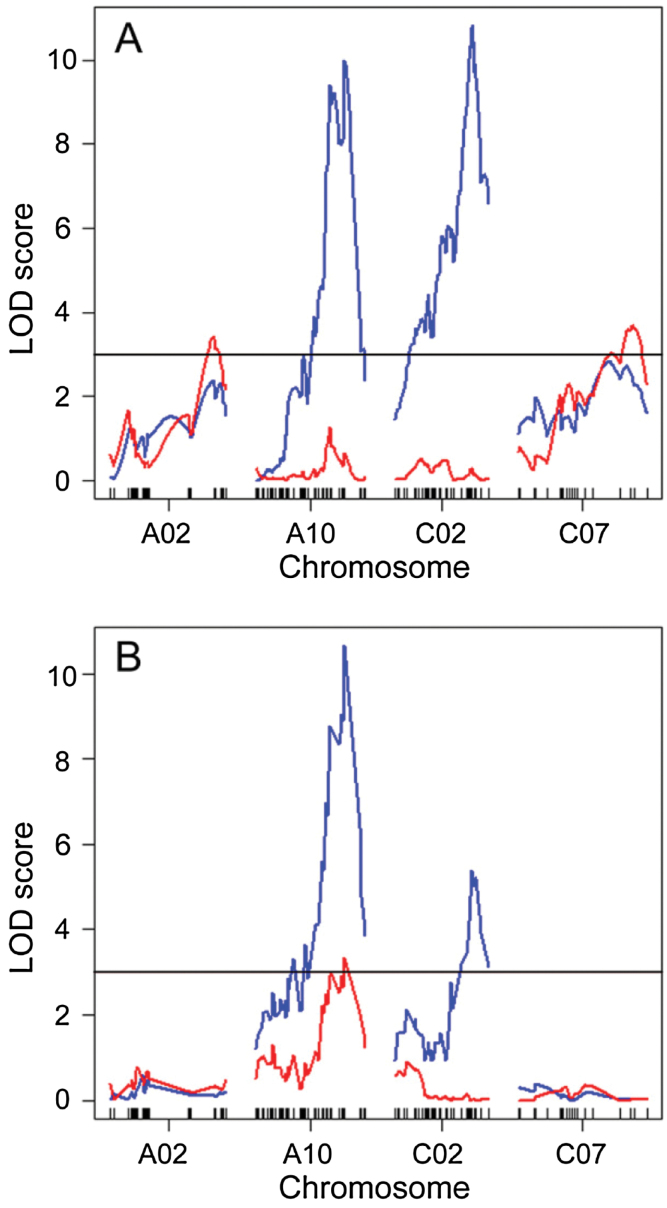
LOD profiles comparing conditional (incorporating DTF as a covariate; red) and unconditional (no covariate; blue) QTL scans in the wet (A) and dry (B) environments. The horizontal line indicates the LOD threshold based on 1000 permutations.

Conditional genome-wide scans for RPF in the wet treatment identified a new QTL on A08 (*RPF.*wet4). Interestingly, the high RPF allele at *RPF.wet4* which did not affect flowering time originated from the spring parent, IMC106RR. Another QTL was mapped to the same location on C07 as *RPF.wet3*, identified previously in the unconditional scan (Fig. 5A). These findings further support a genetic basis to RPF that does not entirely overlap with that of DTF. The stepwise model selection used included the DTF term (1.09 Kg ± 0.08) which resulted in the disappearance of *RPF.wet1* on A10 and *RPF.wet2* on C02. This suggests that the QTLs at these two loci may have been affecting RPF as a downstream result of their effect on DTF (Fig. 1C) in this environment.

The conditional QTL scans of the dry treatment yielded a model with a single QTL on A10 (Fig. 5B) co-localizing in the same location on A10 as *RPF.dry1* (0.50 Kg ± 0.09), along with a DTF effect. The significant impact of this QTL and the DTF covariate term in the model support a mode of causality similar to that of Fig. 1D where the QTL affects RPF directly as well as indirectly through its impact on flowering time.

### Single marker analysis of RPF using models conditional on DTF strata account for a right-censored distribution and further support the direct role of *RPF.dry1* on both traits

In the dry treatment, 22 DH lines were censored (omitted) from the flowering time distribution because they did not flower and, therefore, had no observed flowering time to use as a covariate in the conditional QTL analysis. To account for these missing data, the population was stratified into five classes of approximately 45 lines based on their flowering times (Supplementary Table S4). Single marker analyses of RPF, conditional on DTF strata, were then performed at each of the QTLs identified previously.

**Fig. 6.**
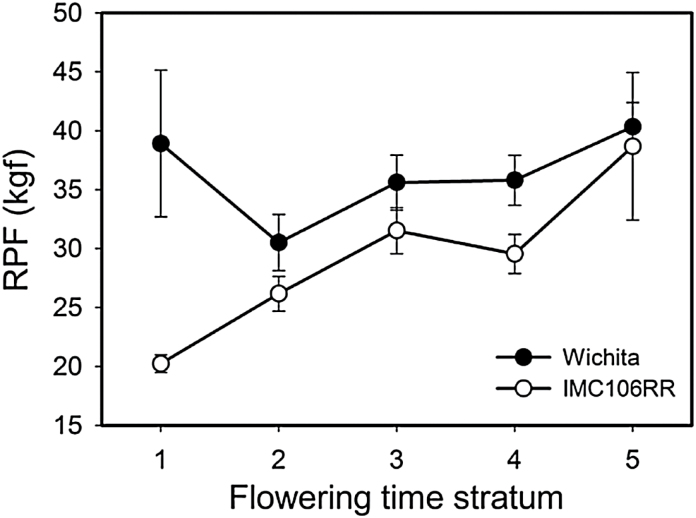
Dependence of RPF in the dry treatment on flowering time strata (1, earliest; 5, latest) for each allele at RPF.dry1 (mean ± SE). See Supplementary Table 4 for further description of the strata.

The stratification factor was highly significant (*P* < 0.0001) in all analyses, further supporting the strong effect of flowering time on RPF. In the dry treatment, only *RPF.dry1* (A10) remained significant as the estimated difference in the mean allele value changed only slightly between the conditional and unconditional analyses (Table 2). In contrast, *RPF.dry2* (C02) became insignificant in the conditional analysis despite the major difference in mean allele values estimated during the unconditional examination (Table 2). Analyses of the wet treatment provided further support for the presence of *RPF.wet3* (C07) and *RPF.*wet4 (A08) and produced an insignificant result for *RPF.wet2*. *RPF.wet1* remained significant in the conditional model suggesting its effect may be constitutive across treatments. To further illustrate that the alleles at *RPF.dry1* affect roots independently of flowering time, mean RPF values were plotted as a function of DTF strata, where it is demonstrated that RPF values are higher for the Wichita allele across any of the five DTF strata than they are for the IMC106RR allele (Fig. 6).

**Table 2.** Mean RPF difference between parental alleles (Wichita – IMC106RR) estimated in unconditional and conditional (using DTF as a covariate) single marker analyses^a,b^

QTL	Chr	Scan	*RPF.wet* (Kgf)	*RPF.dry* (Kgf)
4	A08	Unconditional	–4.74^a^	–1.69
		Conditional	–4.57^a^	–1.47
1	A10	Unconditional	13.8^b^	10.07^b^
		Conditional	6.80^b^	5.29^b^
2	C02	Unconditional	12.88^b^	7.49^b^
		Conditional	3.28	–0.28
3	C07	Unconditional	5.34^a^	–0.58
		Conditional	6.68^a^	–0.10

^a^ Differences significant at *P* < 0.05.

^b^ Differences significant at *P* < 0.01.

### The effect of *RPF.dry1* is validated in a second field experiment and determined to be acting on taproot size

To validate the effects of *RPF.dry1*, 20 lines representing each parental haplotype at the QTL were selected and the experiment was repeated. The haplotype was defined by the interval spanning the length of the LOD 1.5 confidence interval, a region encompassing a minimum of 1.0Mb (physical positions 13 498 846 to 14 558 300) as estimated by the physical locations of the flanking markers (Cheng *et al.*, 2012) relative to the *B. rapa* reference genome V1.5 (Cheng *et al.*, 2011; Wang *et al.*, 2011).

The pleiotropic effect of *RPF.dry1* was confirmed, as lines carrying the Wichita haplotype flowered an average of 12 days later (*P* < 0.0001) and required nearly 17 Kgf more force to remove the roots (*P* < 0.0001) than lines carrying the IMC106RR haplotype. Conditional analyses accounting for DTF estimated a significant haplotype effect (*P* < 0.05), confirming that the effect of genotype on RPF at this locus is significant even after accounting for DTF.

In this experiment, the root system was harvested after RPF measurement and analysed in an effort to gain a better understanding of the root qualities measured by RPF. RPF was most highly correlated with total root dry mass but had significant correlations with DTF, SFW, taproot dry mass, lateral dry mass, taproot diameter, taproot length and branching zone length (Table 3). No significant correlation was found between RPF and the number of coarse secondary roots. Analyses of the specific root components found the effect of haplotype was significant for all traits except lateral root dry mass, branching zone length, and the number of coarse secondary roots.

**Table 3.** Genetic correlation coefficients of traits measured in the 2012 field experiment (*n* = 39)^a–d^

	DTF	SFW	DMT	DML	DM	TRD	TRL	BZL	NSR
**RPF**	0.40^a^	0.71^c^	0.76^c^	0.82^c^	0.88^c^	0.76^c^	0.67^c^	0.64^c^	0.22
**DTF**		0.48^b^	0.31	0.26	0.30	0.40^a^	0.30	0.28	0.14
**SFW**			0.60^c^	0.70^c^	0.73^c^	0.63^c^	0.38^b^	0.40^b^	–0.05
**DMT**				0.62^c^	0.83^c^	0.71^c^	0.71^c^	0.63^c^	0.21
**DML**					0.95^c^	0.82^c^	0.52^b^	0.52^b^	0.05
**DM**						0.86^c^	0.65^c^	0.61^c^	0.12
**TRD**							0.55^b^	0.50^b^	0.14
**TRL**								0.88^c^	0.41^b^
**BZL**									0.52^b^

^a^ Correlations significant at *P* < 0.05.

^b^ Correlations significant at *P* < 0.01.

^c^ Correlations significant at *P* < 0.0001.

^d^ RPF, root pulling force; DTF, days to flower; SFW, shoot fresh weight; DMT, dry mass taproot; DML, dry mass laterals; DM, dry mass; TRD, tap root diameter; TRL, tap root length; BZL, branching zone length; NSR, number of coarse secondary laterals.

Examination of the correlation matrix reveals significant and generally strong correlations between SFW and all measured root traits except the number of coarse secondary roots. Since aboveground biomass is expected to have a significant association with belowground biomass, the data were re-analysed using models incorporating SFW and DTF as covariates to further investigate the relationship between genotype and the measured root traits while accounting for these correlated and potentially confounding factors. We found that only taproot dry mass was significant in models conditioning on SFW as well as those incorporating both SFW and DTF as covariates (Table 4). It is remarkable that any trait remained significant after conditioning on two correlated traits; this suggests that the specific root trait which this locus is acting upon may be taproot size, as lines with the IMC106RR allele had an average taproot mass 74% as large as those with the Wichita allele. Thus, evidence for the direct effect of this QTL on taproot size provides a more detailed understanding of the genetics and specific root characteristics underlying the observed DTF:RPF correlation and the inferred trade-offs between adaptive drought strategies.

**Table 4.** *F*-values for haplotype (genotype at *RPF.dry1*) in models incorporating DTF and SFW as covariates^a–c^

Trait	Haplotype	Haplotype + DTF	Haplotype + SFW	Haplotype + DTF + SFW
DMT	14.22^b^	10.76^b^	4.81^a^	5.93^a^
DM	6.62^a^	3.88	0.16	0.54
TRD	8.11^b^	3.04	1.22	0.60
TRL	4.69^a^	1.58	1.32	0.52

^a^ Differences significant at *P* < 0.05.

^b^ Differences significant at *P* < 0.01

^c^ DTF, days to flower; SFW, shoot fresh weight; DMT, dry mass taproot; DM, dry mass; TRD, tap root diameter; TRL, tap root length.

## Discussion

### Strong genetic correlations and conditional QTL models indicate that the trade-off between drought escape and avoidance may be due to pleiotropy

The strong correlations observed among root traits, flowering time, and yield in this study are concordant with previous research (Bolaños and Edmeades, 1993; Mitchell-Olds, 1996; Lou *et al.*, 2007; Shi *et al.*, 2009). Our QTL results provide first steps toward understanding the common and independent genomic regions contributing to variation in each of these traits, thus providing a better understanding of their inheritance and the genetic architecture of their covariance. Further, these results suggest a trade-off between drought escape and avoidance strategies as there was a significant difference in yield between early-flowering lines with small root systems and late-flowering lines with larger root systems (Fig. 2).

Co-localization of QTLs discovered through mapping approaches can be considered circumstantial evidence for pleiotropy (Lebreton *et al.*, 1995; Tuberosa *et al.*, 2003; Lanceras *et al.*, 2004). Our results show that RPF and DTF are not invariably linked as four of 12 QTLs show independent effects. Most QTL results in our study support a model of broad-sense pleiotropy (i.e. an allele affecting more than one trait) underlying the correlations we observed between RPF, DTF, and yield. The overall prevalence of genome-wide pleiotropy is expected to be rare, but those genes demonstrating higher levels of pleiotropy (i.e. affecting a larger number of traits) are also expected to have larger effects on a per-trait basis (Wang *et al.*, 2010). Therefore, we should expect that the effect sizes of pleiotropic genes should be larger than those due to genetic linkage. This is consistent with our QTLs on A10 and C02 which showed larger effects, explained more variation on a per trait basis, and had higher LOD support than the other QTLs we discovered. However, further work to create and phenotype near-isogenic lines (NILs), mutants, and transgenics will be necessary to conclusively rule out genetic linkage (Huang *et al.*, 2013; Lovell *et al.*, 2013; Uga *et al.*, 2013). For breeding, this information would enable the design of breeding schemes to dissociate trait covariance should an increase or decrease in root investment be of value to the target production geography. For natural selection, it would facilitate our understanding of the genetics of adaptation in natural populations since pleiotropic genes have been shown to have both adaptive (Le Corre *et al.*, 2002; Toomajian *et al.*, 2006; Lovell *et al.*, 2013) and maladaptive (Rose, 1982; Scarcelli *et al.*, 2007) consequences.

In an effort to elucidate the functional pathway, we utilized the highly correlated, and putatively upstream, flowering time trait as a covariate in conditional analyses, essentially scanning for the significance of genetic effects using residual variation that is not explained by the correlated trait (Broman and Sen, 2009). These results provide support for a model where *RPF.dry1* impacts RPF directly. The clear difference between the effect of the parent alleles at *RPF.dry1* (Fig. 6) demonstrate that the Wichita allele increases RPF regardless of flowering date. In contrast, *RPF.dry2* appears to work indirectly through flowering time since RPF does not differ between alleles when flowering time is used as a covariate. The mean difference in RPF between alleles at the C02 locus may therefore be simply due to the fact that the majority of lines carrying the Wichita allele also flower later. The results of the 2012 QTL validation study suggest that the morphological characteristic underlying RPF may be taproot size as lines carrying the Wichita allele were consistently larger when analyzing the data using conditional models accounting for the correlated traits DTF and SFW.

The proposed mechanism of direct pleiotropy suggests that targeting root-specific promoters might be an avenue for increasing root biomass without major effects on flowering time. However, root-specific reductions in cytokinin, a negative regulator of root system size, were shown to increase root biomass with minimal impacts on shoot growth except that bolting and flowering were delayed (Werner *et al.*, 2010). These results may be indicative of inherent root-to-shoot feedback that would override the efficacy of such a strategy.

### Discovery of root QTLs independent of flowering time QTLs suggest that root system size can be increased without impacts on flowering time

Despite the strong correlation between DTF and RPF across the population, trait values in some DH lines were contrary (i.e. high RPF and early flowering) to this expectation. Accordingly, we mapped two QTLs in the wet environment located on linkage groups A08 and C07, loci which do not co-localize with flowering time QTLs. The IMC106RR allele at the A08 QTL increases RPF, a result opposite to the rest of the QTLs for RPF in which the Wichita allele increases RPF. This result partially explains the transgressive segregation we observed for RPF where some lines required more than 1.5 times more force than Wichita for root removal. Associations between loci on C07 and root traits such as root length and root mass have been identified in other experiments conducted to understand the genetics of nutrient use efficiency (Hammond *et al.*, 2009; Yang *et al.*, 2010; Yang *et al.*, 2011; Shi *et al.*, 2012). Because the markers used in those studies do not overlap with ours, it is difficult to draw strong conclusions about specific locational overlap but it suggests that this chromosome is a source of interesting variation in root biology across *Brassica* species. These QTLs and their associated markers could be valuable resources for breeding larger root systems without correlated responses in maturity.

Many mutants and QTLs associated with root development have been identified in research using the model plant *Arabidopsis thaliana* (Benfey *et al.*, 2010). In particular, several QTLs related to root growth have been mapped to the top of *Arabidopsis* chromosome 1 (Kobayashi and Koyama, 2002; Reymond *et al.*, 2006; Sergeeva *et al.*, 2006; Kellermeier *et al.*, 2013) and the bottom of chromosome 4 (Loudet *et al.*, 2005; Fitz Gerald *et al.*, 2006; Reymond *et al.*, 2006; Kellermeier *et al.*, 2013). The QTLs we identified for RPF on A08 and C07 appear to be in regions of the *B. napus* genome that are homologous to these segments of chromosomes 1 and 4, respectively (Cheng *et al.*, 2011; Zhao *et al.*, 2013). This may be suggestive of root-specific genetic mechanisms that have been conserved within the Brassicaceae, but more research is clearly necessary to confirm this.

### Many candidate genes exist across the five identified QTL regions

Flowering time QTLs have been identified previously on A02, A03, A10, C02, and C03 in other *B. napus* and *B. rapa* populations (Osborn, 1997; Schranz *et al.*, 2002; Osborn and Lukens, 2003; Udall *et al.*, 2006; Long *et al.*, 2007; Shi *et al.*, 2009) and, with the exception of the locus on C03, entirely agree with our results. All of these chromosomal regions are syntenic to the top of *Arabidopsis* chromosome 5 (Parkin *et al.*, 2005), a region that contains the well characterized flowering time genes *CO* (Putterill *et al.*, 1995), *FY* (Simpson *et al.*, 2003), and *FLC* (Michaels and Amasino, 1999), among others. Previous research (Schranz *et al.*, 2002; Razi *et al.*, 2008) as well as the draft genomes of *B. rapa* and *B. oleracea* (Cheng *et al.*, 2011; Cheng *et al.*, 2012; Zhao *et al.*, 2013) indicates that *FLC* has been retained after two rounds of whole-genome duplication (Tang *et al.*, 2012) and is present on all of the aforementioned chromosomes. In addition, *CO* has been maintained on A02, A10, and C02; and *FY* on A02 and A03. Beyond the three relatively well characterized genes, discussed above, examination of the draft A and C genomes suggests that ~22 additional genes with gene ontology (GO) annotations to flowering are predicted to reside within these intervals.

Several studies in species of *Brassica* have mapped QTLs for flowering time which overlap with those for primary root (taproot) traits such as fresh weight, length, and width (Lou *et al.*, 2007; Lu *et al.*, 2008; Kubo *et al.*, 2010; Yang *et al.*, 2010). In agreement with the results of our study, at least one of the QTLs identified in each of those experiments was in a location orthologous to the top of *Arabidopsis* chromosome 5. Approximately 20 genes with GO annotations to roots are predicted to lie within this region. We hesitate to suggest any of them as primary candidates since the genetics of root development are poorly understood and their suggestion would be entirely speculative. The results of our conditional analyses also suggest that we should consider the possibility that a flowering time gene may be acting pleiotropically. For instance, it was recently shown that the protein product of the transcription factor *FLC* has over 500 potential binding sites in the *Arabidopsis* genome, sites which were enriched in several GO categories including response to stress and abiotic stimulus (Deng *et al.*, 2011). This may be considered circumstantial support for the results of our conditional examinations of the QTL on A10, and its putatively direct role in root development, since it seems possible that *FLC* could be regulating genes involved in root biosynthesis in *trans*. Similar to our results, a recent analysis of *FLC* in *Arabidopsis* found that it impacted leaf shape and trichome number independently of its impact on flowering time (Willmann and Poethig, 2011).

The results of this research support a body of evidence in which traits relevant to differential drought coping strategies may be genetically constrained, thereby creating an inherent trade-off (Mitchell-Olds, 1996; McKay *et al.*, 2003; Heschel and Riginos, 2005; Wu *et al.*, 2010; Franks, 2011). These results must be considered in the context of the *Brassica* species in which little work has been conducted on drought coping mechanisms and none has focused on the roots. We are currently developing NILs so that the many alleles residing within *RPF.dry1* and *RPF.dry2* can be separated and their impacts on root mass and flowering time may be unequivocally estimated. Additionally, these NILs should be grown under diverse growing conditions and different geographies to understand the role of the environment on these traits and its interaction with the underlying genetics. Results from these experiments will inform fine-mapping activities aimed at cloning the causal variant(s), a process that will require identification of many more molecular markers within the candidate QTL regions. This task will be greatly enabled by the recent release of draft *Brassica* genomes (Cheng *et al.*, 2011; Wang *et al.*, 2011; Zhao *et al.*, 2013; Chalhoub et al., 2014). These activities will show whether the QTL co-localization observed in this study is the result of pleiotropy or genetic linkage, ultimately improving our understanding of the genetics of drought physiology and enabling breeding for drought adaptation.

## Supplementary material

Supplementary data can be found at *JXB* online.

Supplementary Table S1. ANOVA results for DTF, RPF, and yield.

Supplementary Table S2. Genetic and phenotypic correlations among traits and environments.

Supplementary Table S3. Detailed summary of all QTLs discovered.

Supplementary Table S4. Flowering time strata used in single marker analyses.

Supplementary Figure S1. Visual representation of the genetic map of the SE-1 doubled haploid population.

Supplementary Data
