# Supplementary material for: QTL analysis of root morphology, flowering time, and yield reveals trade-offs in response to drought in Brassica napus
Source: J Exp Bot. 2014 Nov 4;66(1):245–56. doi: 10.1093/jxb/eru423 (PMC4265167; doi:10.1093/jxb/eru423)
Supplement: Supplementary Data [file supp_66_1_245__index.html]

QTL analysis of root morphology, flowering time, and yield reveals trade-offs in response to drought in Brassica napus — QTL analysis of root morphology, flowering time, and yield reveals trade-offs in response to drought in Brassica napus — Supplementary Data 

# QTL analysis of root morphology, flowering time, and yield reveals trade-offs in response to drought in *Brassica napus*

## Supplementary Data

Data files

**Files in this Data Supplement:**

- Supplementary Data - Supplementary Data
