# Supplementary material for: QTL analysis of root morphology, flowering time, and yield reveals trade-offs in response to drought in Brassica napus
Source: J Exp Bot. 2014 Nov 4;66(1):245–56. doi: 10.1093/jxb/eru423 (PMC4265167; doi:10.1093/jxb/eru423)
Supplement: Supplementary Data [file supp_eru423_jexbot128116_file001.pdf]

Supplementary Material

QTL analysis of root morphology, flowering time and yield and their plasticity to drought in Brassica napus

Richard S. Fletcher, Jack L. Mullen, Annie Heiliger, John K. McKay

Figure S1. Genetic map of the SE-1 doubled haploid population

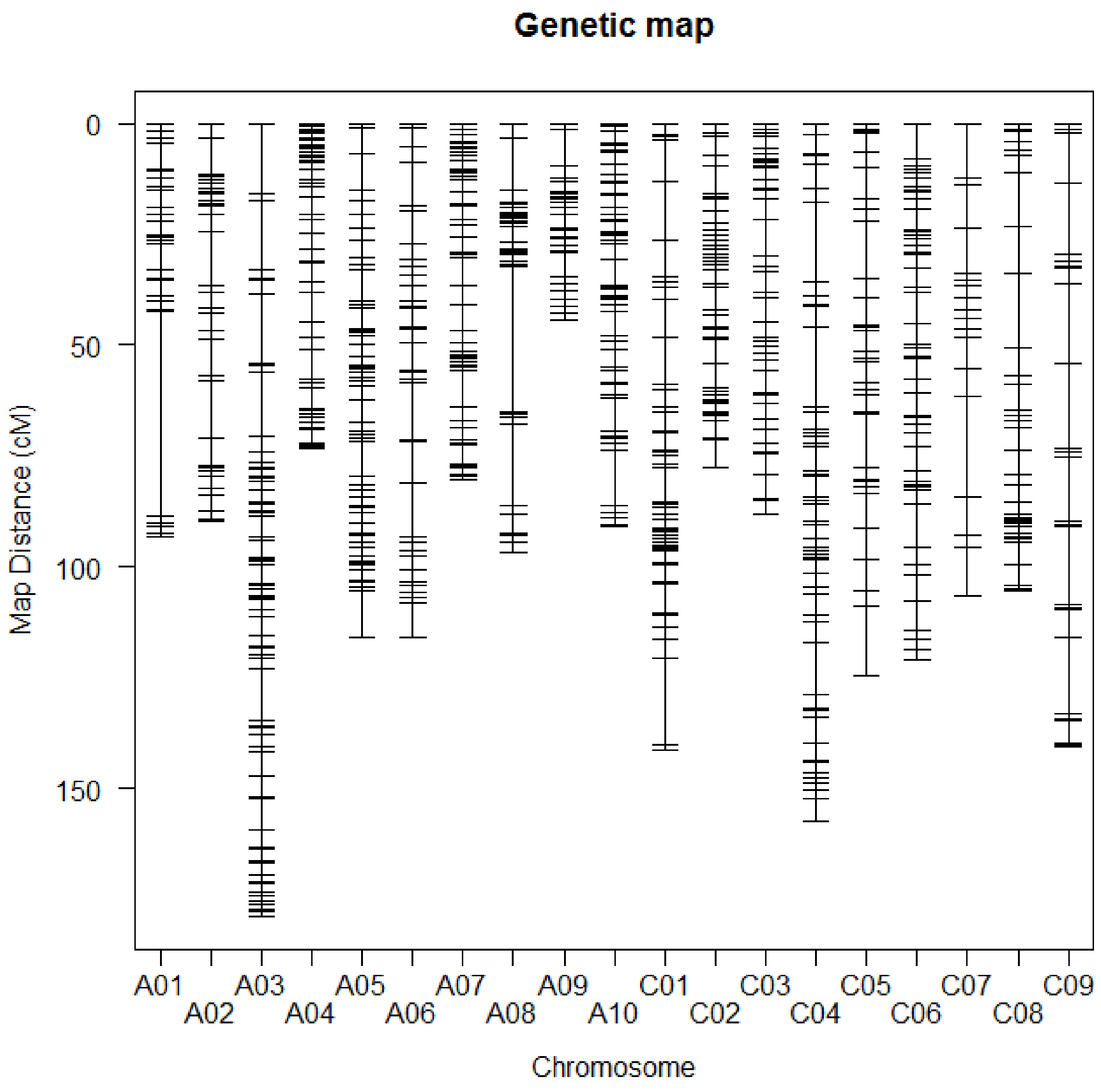

Table S1. ANOVA results for Days to Flower (DTF), Root Pulling Force (RPF), and Yield

	DTF		RPF		Yield	
	F-Value	P	F-Value	P	F-Value	P
DH	31.3	<0.0001	2.7	<0.0001	2.4	<0.0001
Treatment	23.2	<0.0001	40.8	<0.0001	469.0	<0.0001
Treatment (Rep)	17.9	<0.0001	2.1	0.08	18.8	<0.0001
DH x Treatment	1.6	<0.0001	1.0	0.33	1.5	<0.0001

Table S2. Genetic and phenotypic correlations among traits measured in Fort Collins, CO in 2010. Genetic correlations are shown above the diagonal, while phenotypic correlations are below, population sizes used in estimating each correlation coefficient are in parentheses. All correlations are significant at P < 0.0001

	RPF_{dry}	DTF_{dry}	$Yield_{dry}$	RPF_{wet}	DTF_{wet}	$Yield_{wet}$
RPF_{dry}		0.48 (n=195)	-0.41 (n=225)	0.46 (n=225)	0.52 (n=225)	-0.36 (n=225)
DTF_{dry}	0.31 (n=544)		-0.69 (n=195)	0.52 (n=195)	0.89 (n=195)	-0.49 (n=195)
$Yield_{dry}$	-0.23 (n=650)	-0.55 (n=544)		-0.45 (n=225)	-0.68 (n=225)	0.52 (n=225)
RPF_{wet}					0.66 (n=225)	-0.49 (n=225)
DTF_{wet}				0.51 (n=643)		-0.61 (n=225)
$Yield_{wet}$				-0.27 (n=651)	-0.48 (n=643)	

Table S3. Summary of QTL identified with a genome-wide significance threshold of P ≤ 0.05 based on 1000 permutations

Phenotype	Treatment	QTL	Linkage Group	Position (cM)	LOD	R^2	Additive Effect (Wichita allele)
DTF	Wet	*DTF.wet1*	A02	15.4	4.01	2.21	-1.41 ± 0.33
		DTF.wet2	A03	102.0	8.11	4.67	2.04 ± 0.32
		DTF.wet3	A10	75.0	37.69	30.05	5.10 ± 0.32
		DTF.wet4	C02	65.0	41.43	34.50	5.48 ± 0.32
	Dry	*DTF.dry1*	A03	104.2	4.19	3.92	1.62 ± 0.37
		DTF.dry2	A10	75.0	24.19	28.97	4.62 ± 0.38
		DTF.dry3	C02	66.0	33.58	45.51	5.56± 0.37
RPF	Wet	*RPF.wet1*	A10	73.6	13.73	18.46	6.55 ± 0.80
		RPF.wet2	C02	63.3	14.78	20.09	6.75 ± 0.80
		RPF.wet3	C07	95.0	3.34	4.02	3.21 ± 0.85
	Dry	*RPF.dry1*	A10	76.0	12.15	19.71	5.32 ± 0.66
		RPF.dry2	C02	63.3	7.22	11.12	3.92 ± 0.65
Yield	Wet	*Yld.wet1*	A03	85.4	3.14	4.42	-6.78 ± 1.77
		Yld.wet2	A10	74.0	10.17	15.40	-12.84 ± 1.80
		Yld.wet3	C02	64.0	10.95	16.72	-13.23 ± 1.78
	Dry	*Yld.dry1*	A03	141.7	3.12	3.82	-1.52 ± 0.40
		Yld.dry2	A10	78.0	15.57	21.73	-3.84 ± 0.42
		Yld.dry3	C02	66.0	12.98	17.62	-3.24± 0.40
	Sensitivity	*YldSens.1*	A10	73.6	5.19	9.00	-8.73 ± 1.75
		YldSens.2	C02	56.0	6.36	11.17	-9.87 ± 1.78

Table S4. Flowering time strata ranges and the number of lines included in each stratification class

Strata	DTF Range	# Lines
1	60.75-69.3	43
2	69.5-74	51
3	74.75-80.5	41
4	81-88.25	45
5	89-DNF	45
